# Editorial Note: The biology and taxonomy of head and body lice—Implications for louse-borne disease prevention

**DOI:** 10.1371/journal.ppat.1014510

**Published:** 2026-08-13

**Authors:** The PLOS Pathogens Editors

The *PLOS Pathogens* Editors issue this Editorial Note to inform readers that the works cited in this article [1] as References 16, 18, and 43 were retracted after the article’s publication date. PLOS considers that these references are not crucial in supporting the relevant statements in [1].
